# Food Reward Alterations during Obesity Are Associated with Inflammation in the Striatum in Mice: Beneficial Effects of *Akkermansia muciniphila*

**DOI:** 10.3390/cells11162534

**Published:** 2022-08-16

**Authors:** Sabrina J. P. Huwart, Alice de Wouters d’Oplinter, Marialetizia Rastelli, Matthias Van Hul, Willem M. de Vos, Serge Luquet, Patrice D. Cani, Amandine Everard

**Affiliations:** 1 Metabolism and Nutrition Research Group, Louvain Drug Research Institute, Walloon Excellence in Life Sciences and BIOtechnology (WELBIO), UCLouvain, Université Catholique de Louvain, 1200 Brussels, Belgium; 2 Laboratory of Microbiology, Department of Agrotechnology and Food Sciences, Wageningen University, 6708WE Wageningen, The Netherlands; 3 Human Microbiome Research Program, Faculty of Medicine, University of Helsinki, 00014 Helsinki, Finland; 4 Unité de Biologie Fonctionnelle et Adaptative, CNRS, Université de Paris, F-75013 Paris, France

**Keywords:** food reward, *Akkermansia muciniphila*, obesity, gut microbiota, food intake, inflammation, lipoprotein lipase

## Abstract

The reward system involved in hedonic food intake presents neuronal and behavioral dysregulations during obesity. Moreover, gut microbiota dysbiosis during obesity promotes low-grade inflammation in peripheral organs and in the brain contributing to metabolic alterations. The mechanisms underlying reward dysregulations during obesity remain unclear. We investigated if inflammation affects the striatum during obesity using a cohort of control-fed or diet-induced obese (DIO) male mice. We tested the potential effects of specific gut bacteria on the reward system during obesity by administrating *Akkermansia muciniphila* daily or a placebo to DIO male mice. We showed that dysregulations of the food reward are associated with inflammation and alterations in the blood–brain barrier in the striatum of obese mice. We identified *Akkermansia muciniphila* as a novel actor able to improve the dysregulated reward behaviors associated with obesity, potentially through a decreased activation of inflammatory pathways and lipid-sensing ability in the striatum. These results open a new field of research and suggest that gut microbes can be considered as an innovative therapeutic approach to attenuate reward alterations in obesity. This study provides substance for further investigations of *Akkermansia muciniphila*-mediated behavioral improvements in other inflammatory neuropsychiatric disorders.

## 1. Introduction

The prevalence of obesity has steadily increased in recent decades. The last report of the World Health Organization (2016) announced that 13% of adults were obese in the world [1]. Excess intake of energy-dense food is often considered as a major factor contributing to obesity [2]. Food intake is controlled by homeostatic regulation at the level of hypothalamic neuronal circuits, based on energy need, and by the food reward system, based primarily on hedonic value [3,4,5]. On the one hand, homeostatic regulation of food intake is mainly integrated by the melanocortin system adjusting energy intake to energy expenditure in response to peripheral signals including leptin, ghrelin, insulin and intestinal hormones (glucagon-like peptide 1 (GLP–1), peptide YY (PYY)) [6]. On the other hand, palatable food that is rich in fat and/or sugar stimulates hedonic, reinforcing and motivational processes of the reward system [7,8]. Dopaminergic neurons in the mesocorticolimbic area of the brain comprise the reward system and are stimulated by palatable food to release dopamine from the ventral tegmental area to the striatum, which includes the nucleus accumbens (Nacc). In 2001, Wang et al. first described a dysregulation of the reward system in obese individuals [9]. Since then, food reward alterations have been broadly proven in the literature in both humans and rodent models [10,11,12]. Different studies have shown that long-term overeating is associated with a decrease in the release of dopamine, a decrease in the expression of dopamine receptors 2 and 1 (*Drd2* and *Drd1*) and an increase in the expression of dopamine transporters (*Dat*) [9,11,13,14,15]. This hypofunctioning of the dopamine pathway leads to altered hedonic and motivational behavior feeding inducing unbiased responses to hedonic food [9,16,17]. Importantly, some human *Drd2* and *Drd4* genes’ polymorphisms are associated with eating disorders [18,19,20]. The TaqIA1 allele polymorphism is associated with a decrease in striatal Drd2 abundance up to 40% [14]. This decrease is correlated with addiction and compulsive behaviors [9,20]. However, to date, the mechanisms driving the desensitization of the reward system associated with obesity remain unknown.

Over the last 20 years, the gut microbiota has emerged as a key regulator of host metabolism, including the hypothalamic regulation of food intake through the gut–brain axis [21,22,23]. In obese individuals, the gut microbiota composition is altered, and gut permeability increases. These changes facilitate the translocation of bacterial components such as lipopolysaccharides (LPS) across the gut barrier into the systemic circulation. The small increase in serum LPS levels observed in obesity is called metabolic endotoxemia [24,25]. LPS activates nuclear factor-kappa B (NF-kB) and c-Jun N-terminal kinase (JNK) inflammatory pathways through toll-like receptor 4 (TLR4) and induces inflammation in several organs, including the brain [26]. Hypothalamic inflammation has been characterized by increased activation of inflammatory pathways, activation of microglia and astrocytes and disruption of the blood–brain barrier (BBB) in both obese humans and rodents [27,28]. During obesity, inflammation affects different regions of the brain and is associated with an increased prevalence of cognitive impairments [29,30]. Therefore, we investigated if inflammation also affects the striatum during obesity and could be associated with reward behavioral alterations. For this purpose, we evaluated the activation of inflammatory pathways, the activation of microglia and astrocytes and the BBB integrity in both lean and DIO conditions.

Our team recently determined the causal role of the gut microbiota in the dysregulation of the reward system in the context of obesity [31]. Using fecal material transplantation from obese mice, we showed that the gut microbiota affects the hedonic components of food intake. One consistent gut microbiota disequilibrium associated with obesity was the decreased abundance of *Akkermansia muciniphila* (*A. muciniphila*). Daily supplementation with this bacterium was previously described as exerting beneficial effects on the prevention of body weight gain and metabolic disorders in rodents and humans [32,33,34]. Importantly, *A. muciniphila* alive exerts its beneficial effects without changing significantly the gut microbiota composition [34]. In addition, *A. muciniphila* modulates inflammation and reduces metabolic endotoxemia, adipose tissue inflammation, insulin resistance and fat-mass gain in rodents fed a high-fat diet (HFD) [32,34]. Therefore, we investigated if *A. muciniphila* would be able to modulate the inflammation in the brain. More precisely, we evaluated the effects of *A. muciniphila* administration on the inflammation at the level of the striatum, implicated in reward responses to food intake, and its effects on behavioral and neuronal alterations of the food reward system in obese conditions.

## 2. Materials and Methods

### 2.1. Mice and Experimental Design

All mouse experiments were approved by the ethical committee for animal care of the Health Sector of the UCLouvain (Université catholique de Louvain) under the specific number 2017/UCL/MD/005. Experiments 1 and 2 follow the guidelines of the local ethics committee and are in accordance with the Belgian Law of 29 May 2013 regarding the protection of laboratory animals (agreement number LA1230314).

#### 2.1.1. Experiment 1

A cohort of 9-week-old specific-opportunistic and pathogen-free (SOPF) male C57BL/6J mice (Janvier laboratories, Le Genest-Saint-Isle, France) was housed in a controlled environment (room temperature of 22 ± 2 °C, 12 h daylight cycle) in groups of two mice per cage, with free access to sterile food (irradiated) and sterile water. Upon delivery, mice were allowed to acclimatize during one week, during which they were fed a control low-fat diet (CT, AIN93Mi, Research Diet, New Brunswick, NJ, USA). Mice were then randomly divided into two groups (40 mice, *n* = 20/group), and fed for 8 weeks with CT or a high-fat diet (HFD, 60% fat and 20% carbohydrates (kcal/100 g) D12492i, Research diet). After 4 weeks of follow-up, the mice were placed in behavioral cages to perform the food preference test and the operant wall test. During operant wall protocol, mice were food-restricted and body weights were maintained at 85% of the initial body weight (before the operant wall procedure), as previously described [31]. The caloric restriction allowed the potentiation of the reward response to the stimulus [35,36].

#### 2.1.2. Experiment 2

A cohort of 9-week-old SOPF male C57BL/6J mice (Janvier laboratories) was housed as described above. Upon delivery, mice underwent an acclimatization period of one week, during which they were fed a CT diet. Mice were then randomly divided into two groups (20 mice, *n* = 10/group), and fed for 8 weeks with a HFD (60% fat and 20% carbohydrates (kcal/100 g) D12492i, Research diet). At the same time point, one group was daily treated at the end of the light phase with *A. muciniphila* MucT by oral gavage at a dose of 2.10^8^ colony-forming unit (CFU) suspended in sterile anaerobic phosphate-buffered saline (PBS, Sigma Aldrich, St. Louis, MO, USA), produced and handled as previously described [34]. The placebo group was orally administered an equivalent volume of sterile anaerobic PBS containing a similar end concentration of glycerol (2.5% vol/vol) (Merck, Darmstadt, Germany). Treatments continued for 8 weeks. After 4 weeks of follow-up, the mice were placed in the behavioral cages to perform the food preference test and the operant wall test. During the operant wall protocol, mice were food-restricted and body weights were maintained at 85% of the initial body weight (before operant wall procedure), as previously described [31]. The caloric restriction allowed the potentiation of the reward response to the stimulus [35,36].

### 2.2. Food Preference Test

Mice were exposed to two diets: CT or a high-fat high-sucrose diet (HFHS, 45% fat and 27.8% sucrose (kcal/100 g) D17110301i, Research diet, New Brunswick, NJ, USA) in Phenotyper chambers (Noldus, Wageningen, The Netherlands). The food intakes were recorded during a 3-h session in the end of the light phase, in satiated state (access to food *ad libitum* before and after the test). The percentage of food preference was calculated based on HFHS intake (g) during the food preference test divided by the total food intake (g) eaten during the food preference test.

### 2.3. Operant Wall Test

The wanting component is linked to the motivation to obtain a reward and was evaluated by an operant wall test as previously described, with some adaptations [7,37,38]. Each session of the test was conducted during the end of the light phase, in operant conditioning chambers (Phenotyper chambers) and analyzed by the provided software (Ethovision XT 14, Noldus, Wageningen, The Netherlands). The mice had intermittent access to an operant wall in their home cages. The operant wall system was composed of two levers and two lights and a pellet dispenser. One lever was arbitrarily designated as active, meaning that pressing on this lever initiated the delivery of a sucrose pellet (5-TUT peanut butter-flavoured sucrose pellet, TestDiet, St. Louis, MO, USA) and was associated with a light on. On the other side, another lever associated with a light off was arbitrarily designated as inactive and would never deliver a reward. Mice were trained for the system twice overnight on a fixed-ration schedule (one lever press on the active lever corresponded to one reward), then underwent 2 sessions of 1 h 30 m. Mice were then shifted to progressive ratio sessions (2 h). The number of lever presses on the active lever to obtain a reward was incrementally increased (*n* + 3) for every pellet.

### 2.4. Tissue Sampling

After 8 weeks of follow-up of the mice, including the last 2 weeks under caloric restriction, the mice were exposed for one hour to sucrose pellets (5-TUT peanut butter-flavoured sucrose pellet) before anesthesia with isoflurane (Forene, Abbott, Maidenhead, UK). This aimed to mimic the conditions of the behavioral tests and stimulate the reward system. During anesthesia, blood was sampled from the portal and cava veins, after which mice were euthanatized by decapitation. Striatum and jejunum were precisely dissected and immediately immersed into liquid nitrogen, then stored at −80 °C for further analysis.

### 2.5. RNA Preparation and Real-Time PCR Analysis

To quantify striatal gene expressions, total RNA was extracted from the striatum using TriPure reagent (Roche, Balea, Switzerland). cDNA was prepared by reverse transcription of 1 µg total RNA using the GoScript Reverse Transcriptase kit (Promega, Madison, WI, USA). Real-time PCR was performed with the QuantStudio 3 real-time PCR system (Thermo Fisher, Waltham, MA, USA). Rpl19 RNA was chosen as the housekeeping gene. All samples were performed in duplicate, and data were analyzed according to the 2^−ΔΔCT^ method. The identity and purity of the amplified product were assessed by melting curve analysis at the end of amplification. Sequences of the primers used for qPCR are available in Supplementary Table S1.

### 2.6. Immunofluorescence

To quantify specific targeted proteins level of microglia and astrocytes (ionized calcium-binding adaptor protein−1 (Iba1) and glial fibrillary acidic protein (Gfap), respectively), at the end of the experiment 1, mice (*n* = 10/group) were anesthetized by isoflurane and transcardially perfused using a solution of cold PBS followed by a solution of cold 4% (w/v) paraformaldehyde (PFA, Merck). The entire brain was carefully harvested, post-fixed in 4% PFA overnight at 4 °C, cryoprotected overnight at 4 °C in a solution of sucrose (Merck) 30% (w/v), subsequently frozen in cold isopentane (VWR, Leuven, Belgium) and stored at −80 °C, as previously described [39]. Twenty micrometers-thick serial coronal cryosections from fixed brains were mounted on SuperFrost Plus slides (Menzel Gläser, Thermo Scientific, Waltham, MA, USA) and kept at −20 °C. For striatum, we harvested 8 serial sections per animal from bregma 0.61 mm to 1.41 mm according to The Mouse Brain in Stereotaxic Coordinates (Paxinos, Franklin) and at least three brain sections per animal were used for quantification. Animals with less than 3 exploitable sections for quantification were excluded. Immunofluorescence was performed using Tyramide-signal amplification (TSA) technology, as previously described [39]. Briefly, after antigen retrieval by heating (2100 Antigen Retriever, Aptum, Southampton, UK) the endogenous peroxidases were inhibited in a solution of MeOH (VWR) with H_2_O_2_ (VWR) 0.1% (v/v). Then the sections were incubated for 45 min in blocking solution (TBS, BSA (Merck) 5%, Tween 20 0.1%) and then incubated overnight with a primary antibody (anti-GFAP 1/10,000, ab5804 from Merck or anti-Iba1 1/500, PA5–27436 from Thermo Fischer, Rockford, IL, USA). After washing, sections were incubated for 1 h with Horse Radish Peroxidase-conjugated secondary antibody (DAKO K4003, Agilent, Santa Clara, CA, USA). The fluorescent signal was amplified using Alexa Fluor 488 Tyramide Reagent (B40953 Thermo Fischer). Finally, nuclei were stained with Hoechst 33342 (H1399 Invitrogen, Waltham, MA, USA). Slides were dehydrated and mounted with Dako Fluorescence Mounting Medium (S302380–2, Agilent, Santa Clara, CA, USA). Fluorescent GFAP scans were obtained using an Oyster scanner (3DHistech Pannoramic P250 Flash III, Budapest, Hungary) and fluorescent Iba1 scans using a Zeiss scanner (Axioscan.z1, Oberkochen, Germany). After a blinding procedure, using ImageJ 1.47 software, the region of interest (ROI) corresponding to the ventral striatum core, shell and striatum dorsal were delimited with white line on each section using The Mouse Brain in Stereotaxic Coordinates as a reference and % of green area were measured. Positive neurons were manually counted within each ROI and a mean value was obtained for each animal. At least three brain sections per animal were considered. Cell countings were double blinded. Quantifications corresponds to the mean of two blinded independent experimenters.

### 2.7. Plasma Multiplex Analysis

Plasma levels of tumor necrosis factor α (TNFa) were measured by multiplex assay kits based on chemiluminescence detection and following manufacturer’s instructions (Meso Scale Discovery (MSD), Gaithersburg, MD, USA). Analyses were performed using a QuickPlex SQ 120 instrument (MSD) and DISCOVERY WORKBENCH^®^ 4.0 software (MSD, Rockville, MD, USA).

### 2.8. Statistical Analysis

Statistical analyses were performed using GraphPad Prism version 9.1.2 for Windows (GraphPad Software, San Diego, CA, USA). Data are expressed as mean ± SEM. Differences between two groups were assessed using an unpaired Student’s *t*-test. In case variance differed significantly between groups according to the Fisher test, a non-parametric (Mann–Whitney) test was performed. The difference between two groups and different time points was assessed using a two-way ANOVA repeated measurement, followed by a Bonferroni post-hoc test. qPCR outliers have been excluded after a Grubbs test.

## 3. Results

### 3.1. Diet-Induced Obese Mice Show Behavioral and Neuronal Alterations in Response to Food Reward

**Figure 1:**
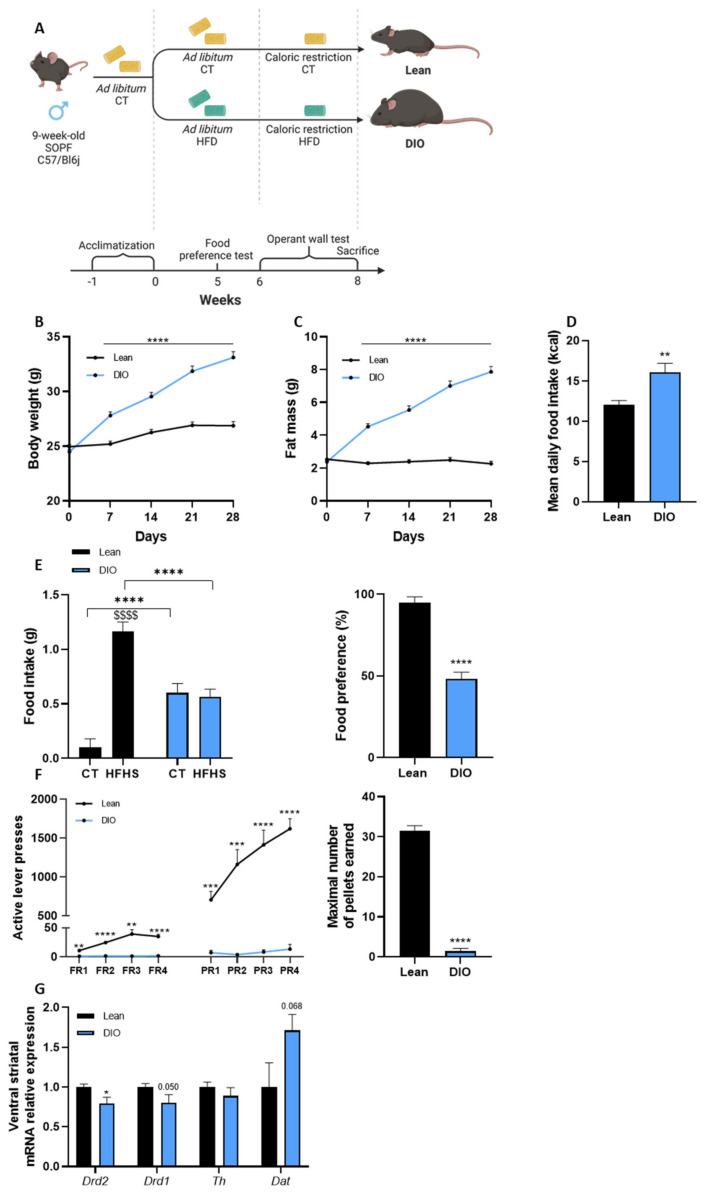
Obesity is associated with an increased body weight, fat mass and calories intake with an alteration of the food reward system. Mice were monitored during 8 weeks of a HFD or a CT. (**A**) Experimental plan of the experiment 1. Created with BioRender.com. (**B**) Body weight evolution and (**C**) fat mass evolution over 4 weeks. (**D**) Mean daily food intake in kcal. (**E**) Food preference test showing HFHS and CT intake in grams and preference for HFHS in percentage after 3 h of test by lean and DIO mice. The percentage of food preference was calculated based on HFHS intake (g) during the food preference test divided by the total food intake (g) eaten during the food preference test. (**F**) Operant conditioning test showing the number of active lever presses during the four progressive ratio (PR) sessions and the maximal number of pellets earned during the PR4 by lean and DIO mice. (**G**) Ventral striatal mRNA relative expression of dopamine receptor 2 (*Drd2*), dopamine receptor 1 (*Drd1*), tyrosine hydroxylase (*Th*) and dopamine transporter (*Dat*) measured by real-time qPCR in lean and DIO mice. Data are shown as mean ± SEM. *p*-values were obtained after two-way ANOVA repeated measure followed by Bonferroni post-hoc test. (*n* = 20/group). (**B**,**C**) after unpaired Student’s *t*-test (*n* = 20/group) (**D**) after two-way ANOVA followed by Bonferroni post-hoc test. (*n* = 10–12/group) (**E**) after two-way ANOVA followed by Bonferroni post-hoc test (*n* = 12/group) (**F**) after unpaired Student’s *t*-test or non-parametric Mann-Whitney test (*n* = 9–12/group) (**E**,**F**,**G**) *: *p*-value < 0.05; **: *p*-value < 0.01; ***: *p*-value < 0.001; ****: *p*-value < 0.0001 between lean vs. DIO. $$$$: *p*-value < 0.0001 between CT vs. HFHS food intake.

Mice were fed during 8 weeks with a CT (lean group) or a HFD to establish obesity (diet-induced obese group (DIO)) (Figure 1A). As expected, after 1 week of HFD diet, DIO mice presented an increase in body weight and fat mass compared to lean mice (Figure 1B,C) and their mean daily food intake in calories was statistically increased (Figure 1D). After 4 weeks of respective feeding, we assessed the liking and wanting components of the food reward system by performing behavioral tests. We first assessed the liking or hedonic component by analyzing the tropism towards palatable food (HFHS) over control food (CT) during a food preference test. A preference for HFHS would suggest a rewarding response specifically to palatable food. As expected, and consistent with the literature [38,40], lean mice showed a great preference for palatable food as shown by the higher intake of HFHS compared to CT (*p* < 0.0001 HFHS vs. CT in the lean group, Figure 1E); in contrast, DIO mice had no preference for HFHS over the CT diet (*p* = 0.77 HFHS vs. CT in the DIO group, Figure 1E). Importantly, DIO mice ate more than 2-fold less HFHS than lean mice (*p* < 0.0001 DIO vs. lean mice, 1.16 g vs. 0.57 g, respectively). Lean mice presented a higher preference for palatable food than obese mice (*p* < 0.0001 DIO vs. lean mice, Figure 1E).

We next assessed the motivational component of the food reward by subjecting the mice to an operant conditioning task in which their eagerness to obtain rewarding food (peanut butter-flavored sucrose pellets) was tested. The mice had to press on a lever to obtain the food reward. Lean and DIO mice were evaluated for incentive motivation on progressive ratio (PR) sessions, which required an increasing number of lever presses to obtain a new sucrose pellet (3 lever presses more for each subsequent reinforcer (r = 3*n* + 3; *n* = reinforcer number)). The PR sessions thereby measured the amount of effort an animal was willing to exert to obtain food rewards and relied on the motivational aspect of the reward system. Compared to lean mice, DIO mice pressed significantly less on the lever to obtain a reward, suggesting a reduction in motivation (Figure 1F). Consistent with the number of lever presses, the breaking point or the number of sucrose pellets earned during a session was significantly lower in the DIO group than in the lean group (Figure 1F).

To complete the behavioral analysis, we further investigated the reward system by analyzing the dopaminergic system in mesocorticolimbic structures of the brain (Figure 1G). The expressions of *Drd2* and *Drd1* were decreased in the ventral striatum of obese mice (*p* = 0.028 and *p* = 0.050, respectively) compared to lean mice, whereas the expression of the *Dat*, which is responsible for the recapture of dopamine, tended to increase (*p* = 0.068, Figure 1G). The expression of the tyrosine hydroxylase (*Th*), the rate-limiting enzyme synthetizing dopamine, was not affected in DIO mice (Figure 1G). Altogether, these behavioral and neuronal analyses suggest a hypofunctioning of the dopaminergic pathways in obese rodents, as already described in the literature.

### 3.2. Obesity Is Associated with Inflammation and Blood–Brain Barrier Alterations in the Striatum

**Figure 2:**
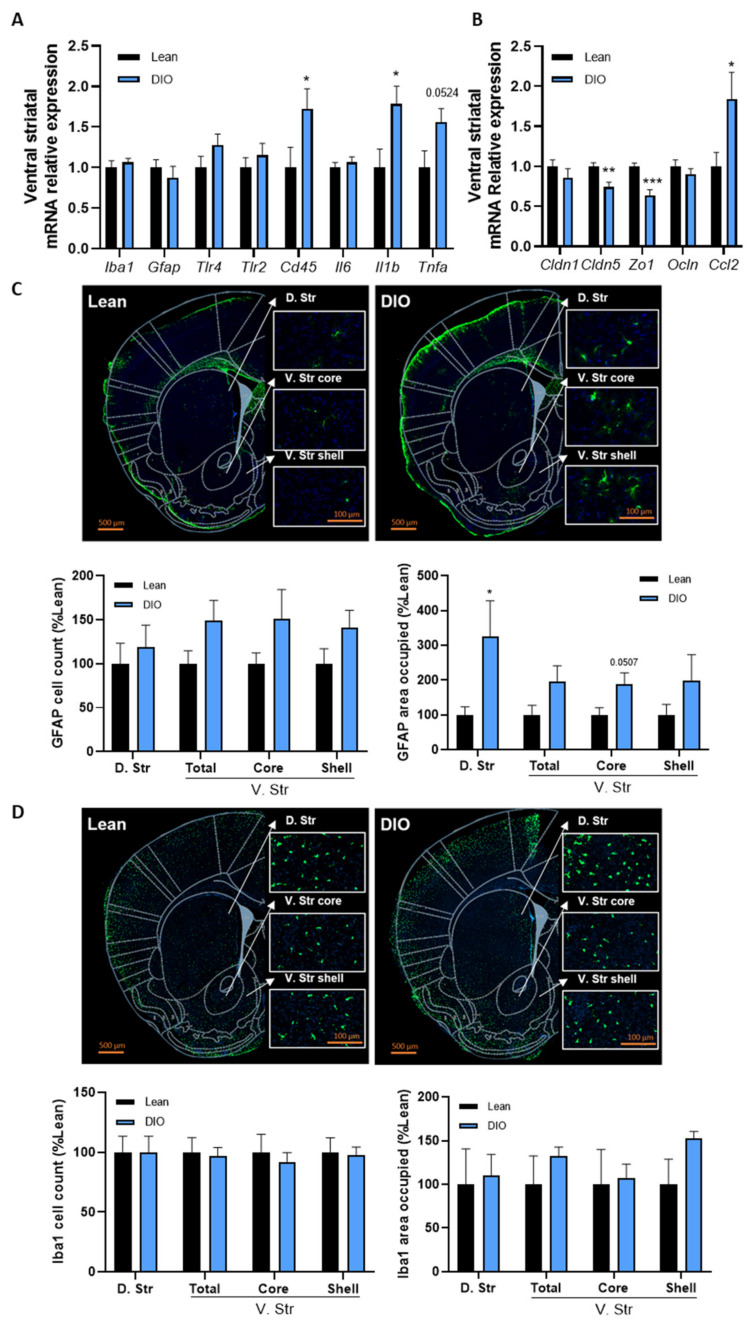
Obesity is associated with inflammation and blood–brain barrier alterations in the striatum. (**A**) Ventral striatal mRNA relative expression of ionized calcium-binding adapter (*Iba1*), glial fibrillary acidic protein (*Gfap*), toll-like receptor 4 (*Tlr4*), toll-like receptor 2 (*Tlr2*) cluster of differenciation 45 (*Cd45*), interleukin 6 (*Il6*), interleukin 1 beta (*Il1b*) and tumor necrosis factor alpha (*Tnfa*) and (**B**) claudin–1 (*Cldn1*), claudin–5 (*Cldn5*), zonula occludens 1 (*Zo1*), occludin (*Ocln*) and C-C chemokine ligand 2 (*Ccl2*) measured by real-time qPCR in lean and DIO mice. (*n* = 9–10/group). (**C**) Representative immunofluorescence of the dorsal striatum (D. Str), the ventral striatum (V. Str) total, core and shell and quantification of both the area occupied by astrocytes cells and the GFAP+ cells in these regions of lean and DIO mice (*n* = 4–5/group). (**D**) Representative immunofluorescence of the dorsal striatum (D. Str), the ventral striatum (V. Str) total, core and shell and quantification of both the area occupied by microglial cells and the Iba1+ cells in these regions of lean and DIO mice (*n* = 5/group). Data are shown as mean ± SEM. *p*-values were obtained after unpaired Student’s *t*-test or non-parametric Mann–Whitney test. *: *p*-value < 0.05; **: *p*-value < 0.01; ***: *p*-value < 0.001 between lean vs. DIO.

We investigated whether the dysregulation of food reward behaviors and dopaminergic pathways during obesity was linked to inflammation in the striatum. Since inflammation in the brain is associated with the activation of microglia and astrocytes, which are associated with an increase in the expression of ionized calcium-binding adaptor protein–1 (*Iba1*) and glial fibrillary acidic protein (*Gfap*), respectively, we analyzed the levels of these markers in the ventral striatum of lean and DIO mice using qPCR. We did not observe any difference in the expression of these markers between lean and obese mice (Figure 2A). We also analyzed the expressions of receptors for some fatty acids and LPS (*Tlr4)* and for a variety of microbial products (*Tlr2*) implicated in inflammation as well as the expressions of infiltrating immune cell markers (cluster of differentiation 45 (*Cd45*) and pro-inflammatory cytokines (interleukin–6 (*Il6*), interleukin–1 β (*Il1b*) and tumor necrosis factor α (*Tnfa*)). We detected significant increases in the expressions of *Cd45* and *Il1b* and an increasing trend for *Tnfa* expression in obese mice as compared to lean mice (*p* = 0.044, 0.025 and 0.052, respectively, DIO vs. lean mice, Figure 2A), suggesting the induction of inflammation in the mesocorticolimbic area by HFD-induced obesity. The striatal expressions of *Tlr4*, *Tlr2* and *Il6* were not significantly changed upon HFD (Figure 2A).

The BBB is essential to protect brain structures from toxins, pathogens and excess immune cell infiltration and to maintain neuronal integrity [41,42,43]. Since inflammation in the CNS is associated with disruption of the BBB in several neurological disorders, including some associated with obesity [44,45], we further analyzed the integrity of the BBB in the striatum by measuring the expression of key tight-junction proteins and the C-C chemokine ligand 2 (*Ccl2*), a regulator of BBB permeability. Interestingly, the expression levels of claudin–5 (*Cldn5*) and zonula occludens 1 (*Zo1*) were significantly decreased in obese mice compared to lean mice (*p* = 0.0033 and 0.0004 DIO vs. lean mice, Figure 2B), whereas claudin–1 (*Cldn1*) and occludin (*Ocln*) levels were not changed (Figure 2B). In addition, the expression of *Ccl2* was particularly increased in DIO mice as compared to lean mice, which can further permit infiltration of immune cells from the systemic circulation (Figure 2B).

To better visualize the potential activation states of astrocytes and microglia, we performed immunohistochemical staining for GFAP and Iba1 in the striatum (Figure 2C,D). Evidence of an inflammatory response in the brain includes modifications in microglia and astrocytes morphologies and numbers, meaning activation [46,47]. We found that DIO induced a significant astrocyte activation in the dorsal striatum as shown by an increased GFAP-labelled area (*p* = 0.049, DIO vs. lean mice, Figure 2C). We also found a trend for an increased GFAP-labelled area in the ventral striatum core, in the DIO group as compared to the lean group (*p* = 0.051, DIO vs. lean mice, Figure 2C), whereas increases in astrocytes number (in cell count) were not significant (Figure 2C). This suggests changes in the morphology of the astrocytes without impacting the number of astrocytes. The changes in morphology of brain cells are often associated with the activation of inflammation pathways and immune cells. Surprisingly, astrocytes were mainly activated in the right dorsal and the ventral striatum, while these regions in the left hemisphere did not present astrocyte activation (Supplementary Figure S1A). Iba1 immunostaining did not show any difference in terms of microglial cell activation between lean and obese mice (Figure 2D and Supplementary Figure S1B).

### 3.3. The Administration of A. muciniphila Improves the Motivational Component of Food Reward Altered by DIO

**Figure 3:**
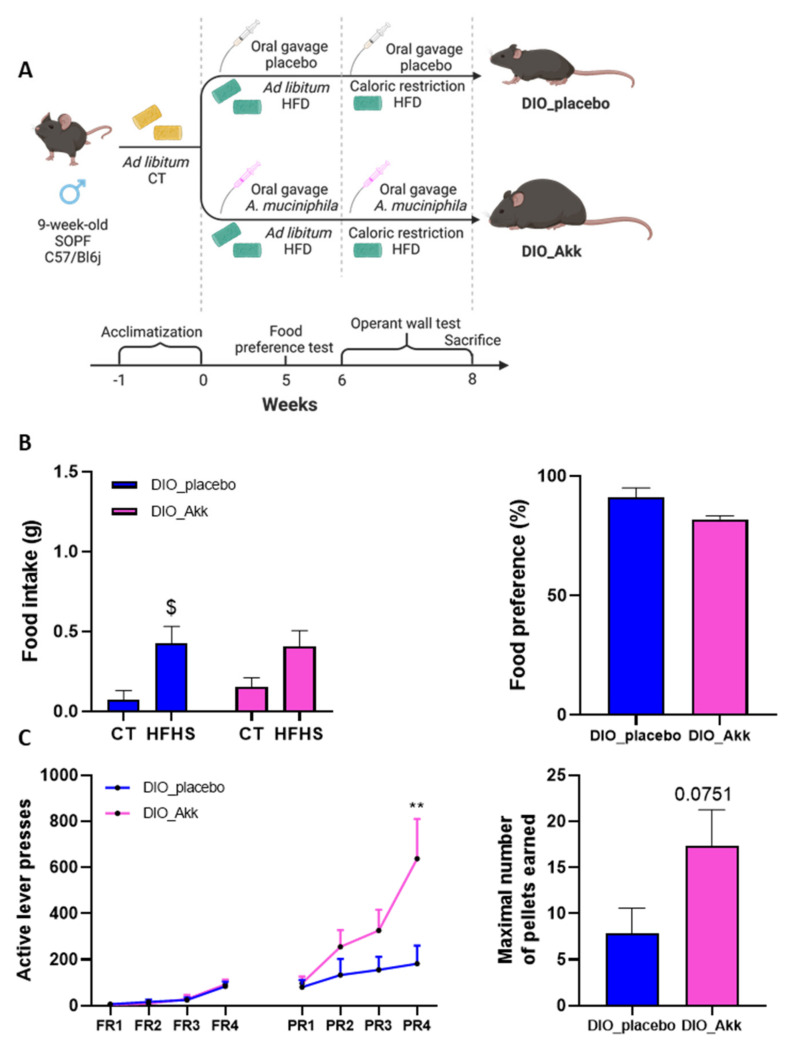
*A. muciniphila* administration improves the motivational component of food reward associated with obesity. (**A**) Experimental plan of the experiment 1. Created with BioRender.com. (**B**) Food preference test showing HFHS and CT intake in grams and preference for HFHS in percentage after 3 h of test by DIO mice treated with placebo (DIO_placebo) and *A. muciniphila* (DIO_Akk). (**C**) Operant conditioning test showing the number of active lever presses during the four progressive ratio (PR) sessions and the maximal number of pellets earned during the PR4 by DIO mice treated with placebo (DIO_placebo) and *A. muciniphila* (DIO_Akk). The percentage of food preference was calculated based on HFHS intake (g) during the food preference test divided by the total food intake (g) eaten during the food preference test. Data are shown as mean ± SEM. *p*-values were obtained after two-way ANOVA followed by Bonferroni post-hoc test. (*n* = 7/group) (**B**) after two-way ANOVA repeated measure followed by Bonferroni post-hoc test (*n* = 6/group) (**C**) after unpaired Student’s *t*-test or non-parametric Mann-Whitney test (*n* = 6–7/group) (**B**,**C**) **: *p*-value < 0.01 between DIO_placebo vs. DIO_Akk. $: *p*-value < 0.05 between CT vs. HFHS food intake.

*A. muciniphila* is a beneficial bacterium that counteracts DIO and metabolic disorders associated with low-grade inflammation [32,33,34]. Therefore, we evaluated the potential effect of *A. muciniphila* administration on the reward system in obese animals (Figure 3A).

During the food preference test, the total HFHS intakes of both DIO groups were similar, and comparable to the amount eaten by the DIO group in experiment 1 (mean = 0.43 g in Figure 3B and mean = 0.56 g in Figure 1E, respectively). This finding confirms the alteration of the hedonic food intake associated with obesity. Obese mice treated with *A. muciniphila* did not show any preference for a specific diet (*p* = 0.24 for the comparison between CT and HFHS in the DIO_Akk group, Figure 3B) or increased food preference percentage as compared to DIO_placebo mice (*p* = 0.0837 between DIO_Akk and DIO_placebo, Figure 3B). This suggests that the administration of *A. muciniphila* did not seem to affect the altered hedonic intake of obese animals.

Since reward-based food intake is characterized by three psychosocial components (i.e., the liking, the wanting and the learning), we decided to further investigate the motivation with an operant conditioning task in DIO mice supplemented with *A. muciniphila*. The PR sessions showed a statistically significant increase in the motivation of the obese mice supplemented with *A. muciniphila* at PR4 compared to obese mice treated with the placebo (*p* = 0.0022 DIO_Akk vs. DIO_placebo groups, Figure 3C). Consistent with the lever pressing parameter, the maximum number of reinforcers obtained tended to be increased in obese mice treated with *A. muciniphila* compared to placebo-treated mice (*p* = 0.075, Figure 3C).

### 3.4. Beneficial Effects of Akkermansia muciniphila on the Reward System Are Associated with a Reduction in Striatal Inflammation and Lpl Expression

**Figure 4:**
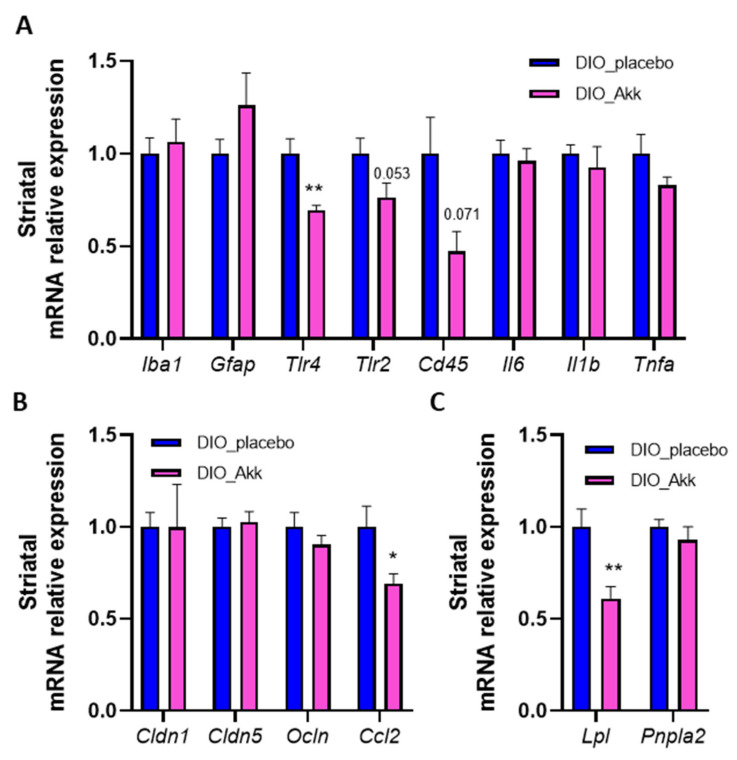
*A. muciniphila* administration reduces mesocorticolimbic markers of inflammation and BBB permeability associated with obesity and lowers striatal *Lpl* expression. (**A**) Striatal mRNA relative expression of ionized calcium-binding adapter (*Iba1*), glial fibrillary acidic protein (*Gfap*), toll-like receptor 4 (*Tlr4*), toll-like receptor 2 (*Tlr2*), cluster of differentiation 45 (*Cd45*) interleukin 6 (*Il6*), interleukin 1 beta (*Il1b*), tumor necrosis factor alpha (*Tnfa*); and (**B**) claudin–1 (*Cldn1*), claudin–5 (*Cldn5*), occludin (*Ocln*), C-C chemokine ligand 2 (*Ccl2*); and (**C**) lipoprotein lipase (*Lpl*) and patatin-like phospholipase domain containing 2 (*Pnpla2*) measured by real-time qPCR in DIO mice treated with placebo (DIO_placebo) and *A. muciniphila* (DIO_Akk). (*n* = 5–10/group). Data are shown as mean ± SEM. *p*-values were obtained after unpaired Student’s *t*-test or non-parametric Mann–Whitney test. *: *p*-value < 0.05; **: *p*-value < 0.01 between DIO_placebo vs. DIO_Akk.

From a mechanistic perspective, the dysregulation of the reward system associated with obesity might be due to inflammation in the striatum (Figure 1 and Figure 2). Based on the ability of *A. muciniphila* to improve the motivational alterations induced by a HFD (Figure 3C), and because systemic anti-inflammatory effects have previously been described for this bacterium, we analyzed several markers of inflammation in the striatum of obese mice supplemented with or without *A. muciniphila* [34]. Importantly, the expressions of pattern recognition receptors *Tlr4* and *Tlr2* and marker of infiltrating immune cells (*Cd45*) were decreased in the striatum of *A. muciniphila*-supplemented mice compared to placebo-treated mice (*p* = 0.0015, *p* = 0.0533 and *p* = 0.0713, respectively, in DIO_Akk vs. DIO_placebo, Figure 4A). The striatal expressions of markers of microglia (*Iba1*) and astrocytes (*Gfap*), as well as pro-inflammatory cytokines (*Il6*, *Il1b*, *Tnfa*), were not changed between the DIO_Akk group compared to the DIO_placebo group (Figure 4A). When investigating the origin of inflammation, we first evaluated the integrity of the BBB. We did not find any change in the expression of key tight-junction proteins *Cldn1*, *Cldn5* and *Ocln*, but we found a decrease in the expression of *Ccl2* in the striatum of *A. muciniphila*-treated mice compared to placebo-treated mice (*p* = 0.0382 in DIO_Akk vs. DIO_placebo, Figure 4B). The decrease in this regulator of BBB permeability could explain the decrease in infiltrating immune cells of the systemic circulation.

Then we measured systemic inflammation by quantifying the plasma levels of TNFa and IL6 in both groups. *Akkermansia*-treated mice showed a decreased systemic TNFa level as compared to the DIO_placebo group, but no change in the concentration of Il6 (Supplementary Figure S2).

Taken together, our results suggest that *A. muciniphila* supplementation reverses motivational alterations associated with obesity, potentially through the modulation of the inflammation in mesocorticolimbic structures.

According to previous studies, a mechanism involved in alterations in behavioral reward and dopaminergic transmission implies central lipid sensing through the lipid-processing lipoprotein lipase (Lpl). Importantly, in our cohort, mice receiving *A. muciniphila* showed a highly significant decrease in *Lpl* expression in the striatum as compared to placebo-treated obese mice (*p* = 0.0058, Figure 4C). Interestingly, a similar increase in motivational performance for food-seeking behavior has been assessed with viral-mediated knockdown of Lpl in mice in a progressive ratio operant conditioning paradigm [7,37]. Another lipid sensor in the brain is the adipose triglyceride lipase (encoded by the *Pnpla2* gene), which catalyzes the rate-limiting step of lipolysis. In our cohort, the expression of *Pnpla2* was not different between DIO mice supplemented with *A. muciniphila* and the placebo (Figure 4C). Therefore, the difference in lipid sensing and the subsequent inflammation seem Lpl-mediated in our context. Importantly, we also assessed the expression of *Lpl* in the jejunum, where the lipid metabolism and absorption mainly take place, but we did not observe any difference in *Lpl* expression between *A. muciniphila* and placebo DIO mice (Supplementary Figure S3). The *Akkermansia*-induced changes in the *Lpl* expression seem specific to the brain.

## 4. Discussion

Alterations in reward processes are main contributors to excessive eating behaviors and play a crucial role in maintaining a healthy body weight. The identification of the mechanisms and factors involved in reward dysregulations associated with obesity is therefore of the utmost importance.

First, we aimed to validate behavioral and neuronal alterations in the reward system of obese animals. From a behavioral perspective, we assessed the liking and the wanting components of the food reward by the food preference and the operant wall tests, respectively. The liking component is related to the pleasure felt by eating a specific rewarding food whereas the wanting component is related to the motivation to obtain it [48,49]. During the food preference test, mice were exposed for the first time to a palatable diet that stimulates the mesocorticolimbic system and induces pleasure. The absence of a preference for palatable food and the reduced intake of this diet in DIO mice suggests a dysregulation of the hedonic component of food intake associated with obesity. These results are consistent with other studies investigating the hedonic component of food intake through exposure to a palatable diet [31,38,40,50] or using a variant of the food preference test, the two-bottle choice test, where obese rodents are less attracted to a sucrose solution [51]. Moreover, the results obtained using an operant conditioning paradigm confirmed the altered motivational reward system associated with obesity: DIO mice pressed less on the lever to obtain food reward than lean mice, as previously reported in the literature [52]. In the brain, obese conditions are associated with modifications in the expression of key dopaminergic markers, such as dopaminergic receptors 1 and 2 and the dopamine transporter. These changes in the transcription of dopaminergic markers suggest hypofunctioning of the dopaminergic pathway and reflect the behavioral dysregulations of liking and wanting components of food reward observed during the food preference and operant conditioning tests.

The increased systemic low-grade inflammatory tone associated with obesity promotes the presence of inflammation in the CNS. For many years, studies have focused on hypothalamic inflammation, which was shown to be involved in the development of the obese phenotype. However, recently, inflammation has been recognized as also affecting other brain regions, such as the cortex or hippocampus, in the context of obesity [27,30,42]. Furthermore, obesity-derived brain inflammation is associated with behavioral and cognitive alterations [29,53,54]. We tested the inflammation hypothesis in the striatum to highlight the mechanisms underlying the altered reward system in obesity. Quantification of inflammatory markers by qPCR and immunofluorescence staining revealed the activation of inflammatory mediators in the mesocorticolimbic area following HFD treatment. More specifically, we documented the upregulation of *Cd45* expression, which reflects the infiltration of immune cells and increased expression of pro-inflammatory cytokines (*Il1b* and *Tnfa*) in the ventral striatum of obese mice. One reason that might explain why we observed an increase in *Cd45* and some pro-inflammatory cytokines mRNA expressions without (yet) *Iba1* increased expression (specific for microglia) would be the sequence of events in the cascade of immune activation in the brain. Indeed, it has been shown that the HFD-induced inflammation is time and region specific [30], so we can hypothesize that the immune infiltration comes before the activation of resident microglia. In addition, it has been demonstrated that HFD-induced inflammation in the brain is not always associated with Iba1 increase [30]. There is a possibility that longer HFD treatment would be needed to observe Iba1 increase [55].

Importantly, this study links inflammation in the striatum with altered hedonic and motivational reward behaviors during obesity. We also showed astrocyte activation, which was detected using GFAP immunolabelling, in the dorsal striatum and in the core of the ventral striatum of obese mice. This activation seemed to be specific to the right striatum, as the left striatum did not show any significant difference in the GFAP-positive area. Further investigations are needed, but the results suggest asymmetric brain pathways. In 2018, Han et al. also evoked asymmetry when they reported that motivation and dopamine activity in the brain are mediated by right, but not left, vagal sensory ganglion activation [56]. GFAP immunolabelling results are consistent with studies suggesting the activation of astrocytes in the cerebellum and hypothalamus during obesity [27,30]. Astrocytes adopt a reactive phenotype with changes in their morphology that alters their functions and impairs their abilities to maintain brain homeostasis. Moreover, astrocytes also contribute to the interaction between inflammation induction and the dopaminergic reward system. Indeed, astrocytes modify synaptic dopamine levels by presenting DAT on their own membrane to degrade dopamine [57]. Therefore, inflammation might be responsible for alterations in food reward and dopamine signaling during obesity.

Inflammation in the CNS may result from local activation of inflammatory pathways but might also be induced by increasing BBB permeability, allowing the translocation of pro-inflammatory mediators from the periphery into the brain. BBB function adapts to stimuli such as pro-inflammatory mediators and is, among others, regulated by astrocytes around microvessels [58]. In obese conditions, the BBB is chronically challenged by pro-inflammatory stimuli and presents increasing permeability, especially in the hypothalamus [41,42,43]. Here, we showed that the BBB was also affected in the striatum in obese animals. We showed decreased expression of the tight-junction proteins Cldn5 and Zo1, as well as increased expression of *Ccl2* in obese mice. Cldn5 is expressed at high levels in the brain, and its loss is associated with neurodegenerative, neuroinflammatory and neuropsychiatric disorders [59]. BBB leakage may result in cytokine and immune cell infiltration in the brain, activating inflammatory pathways [42,43,44,59]. Consequently, high BBB permeability is considered a precursor of CNS inflammation and cognitive deficits. Based on our results, striatal inflammation in obese animals could potentially be due to peripheral pro-inflammatory signals passing through the BBB.

Our team recently showed that the dysregulation of the hedonic component of food intake is transferable by gut microbiota transplantation from obese mice, indicating that the gut microbiota plays a major role in reward dysregulation associated with obesity through the gut–brain axis [31]. Here, we found that the administration of *A. muciniphila* reversed the altered motivation induced by a HFD. *A. muciniphila*-treated mice display an improvement of the number of lever presses during the operant conditioning test. Interestingly, even if our study is the first to show that *A. muciniphila* is able to control food reward behavior, *A. muciniphila* has already been shown to be a beneficial bacterium in the gut–brain axis influencing behavior in other contexts such as in depression-like behavior induced by chronic stress [60], in cognitive impairments associated with obesity [61] as well as in spatial learning and memory in an animal model of Alzheimer’s disease [62]. In our study, the administration of *A. muciniphila* did not seem to restore the altered hedonic intake typical of obesity. However, in a human cohort, *A. muciniphila* abundance was negatively correlated with the food addiction scale [63]. That said, the potential beneficial effects of *A. muciniphila* in the non-obese population still need to be investigated, including in association with different type of diets such as a high fiber diet.

We investigated whether the positive effects of *A. muciniphila* administration on the reward system were associated with an attenuation of the inflammation. qPCR results showed that *A. muciniphila* supplementation in obese mice substantially decreased the expression of *Tlr4* and tended to decrease the expressions of *Tlr2* and *Cd45* in the striatum. TLR2 and TLR4 are key markers of inflammation in the context of obesity since they bind microbial products as LPS or peptidoglycan (PGN) and fatty acids (FAs) that are present at higher levels in the plasma of obese subjects. Then, these receptors trigger inflammation via the NF-kB pathway. Interestingly, Sun, Luquet and Small hypothesized that NF-kB activation directly or indirectly regulates the transcription of the dopamine Drd2 receptors [64]. Therefore, this pathway may be a potential mechanism by which *A. muciniphila* regulates food reward.

In addition, we observed that *A. muciniphila* supplementation in obese mice decreased the systemic inflammatory tone by reducing the plasma level of the pro-inflammatory cytokine TNFa. Circulating cytokines and immune cells of the systemic circulation can cross the BBB, especially when BBB permeability is increased, which we confirmed in the striatum of obese placebo mice. *A. muciniphila* supplementation decreases the expression of *Ccl2*, a regulator of the BBB permeability, which can lead to a decreased leakage of immune cells and cytokines into the brain. Cytokines also activate the NF-kB pro-inflammatory pathway. In this context, the reduction in inflammation markers in the striatum by *A. muciniphila* observed in the present study could also be linked to the systemic effect on TNFa levels. Together, our results suggest that *A. muciniphila* supplementation improves the motivational alterations associated with obesity by modulating CNS inflammatory pathways. Consistent with our results, *A. muciniphila* administration has already been shown to be neuroprotective at the level of the hippocampus in a diet-induced obesity model by modulating cytokine expressions and improving learning and memory processes [65]. Moreover, *A. muciniphila* has been broadly described as an anti-inflammatory bacterium in several pathologies, including obesity (decreasing macrophage infiltration and serum LPS levels), colitis and oral cavity infections [35,66,67].

Finally, qPCR results showed a decrease in the expression of striatal enzyme *Lpl* in obese mice supplemented with *A. muciniphila*. In 2004, Cansell et al. identified this LPL, which is expressed at high levels in the striatum, as a lipid sensor modulating the motivational response to reward [37]. They reported that viral-mediated knockdown of *Lpl* in the ventral striatum to an extent that resembles the level observed with *A. muciniphila* supplementation leads to increased performance on operant conditioning tests for a food response during the progressive ratio conditioning test [37]. Furthermore, the same team recently proved that Lpl expressed in the mesocorticolimbic pathway potentiates the action of circulating lipids and decreases the excitability of striatal Drd2 in medium spiny neurons [7]. Therefore, the reduction in *Lpl* expression and the subsequent reduction in the lipid-sensing ability in the striatum induced by *A. muciniphila* might participate in directly modulating dopaminoceptive signaling and behavioral output, thereby restoring the motivational component observed during the operant conditioning test. Moreover, the potential reduction in FAs levels due to the reduction in Lpl-mediated catabolism of triglyceride (TG) might also participate in the reduction in the expression and activation of the lipid-activated TLR4. Altogether, this potential pathway may be the mechanism by which *A. muciniphila* exerts its beneficial effects on the reward system. Furthermore, both the gut and the brain are fully equipped to detect TG and feedback to the reward system [68]. Hence, *A. muciniphila* supplementation could exert a positive effect on altering both gut TG-sensing ability and vagal feedback to the brain and DA efflux, as reported by Tellez and co-authors [69]. However, we did not observe any modulation of the jejunal expression of *Lpl* in DIO mice supplemented with *A. muciniphila* compared to placebo-treated mice.

Altogether, our results revealed *A. muciniphila* as a novel actor able to improve the dysregulated reward behaviors associated with obesity, potentially through a decreased activation of inflammatory pathways and lipid-sensing ability in the striatum. That said, one of the main limitations in the study design of this second experiment was the absence of a control group of mice fed a control diet. Therefore, even if we demonstrated improvement of the dysregulated reward behaviors associated with obesity with *A. muciniphila* supplementation, one may wonder about its capacity to restore food reward behaviors to lean conditions.

Here, we highlighted CNS inflammation as a mechanism behind reward behavioral dysregulation in obesity as well as dopaminergic hypofunctioning. To go further, an analysis of Drd2 and Drd1 neurons activity in real time during behavioral test could provide additional information. The fiber photometry technique allows this analysis with the real-time fluorescence emitted from a calcium sensor which can be expressed in targeted neurons specifically after genetic modifications. An optic fiber canula must be implanted in targeted regions. To better assess the dopaminergic system in obese condition and after *A. muciniphila* supplementation, other techniques exist such as the quantification of neurotransmitters post-mortem. A real-time quantification of neurotransmitters is also possible thanks to the microdialysis technique. A semi-permeable membrane must be implanted in the region of interest to collect the dialysates.

## 5. Conclusions

This study links the dysregulation of the food reward system with inflammation and alterations in the BBB in mesocorticolimbic areas of obese mice. Our data also identify beneficial effects of *A. muciniphila* administration on the brain reward system by reversing the motivational component of food reward altered by DIO. Moreover, we provide a potential mechanism to explain this effect through a decrease in inflammatory pathway activity and lipoprotein lipase expression. Therefore, our results suggest that *A. muciniphila* supplementation might represent an additional and innovative approach to restore food reward behavior and attenuate striatal inflammation in obese individuals. Finally, this study provides an opportunity for further investigations of the effect of *A. muciniphila* on improving behavior in individuals with other neuropsychiatric disorders associated with CNS inflammation and eating disorders, such as Parkinson’s disease or Alzheimer’s disease.

## Data Availability

The data presented in this study are available on request from the corresponding authors.

## Supplementary Materials

The following supporting information can be downloaded at: https://www.mdpi.com/article/10.3390/cells11162534/s1, Figure S1: Details of immunofluorescence quantification; Figure S2: *Akkermansia muciniphila* administration reduces systemic inflammation; Figure S3: *Akkermansia muciniphila* administration does not modify *Lpl* expression in the jejunum; Table S1: Primer sequences.

## Patents

P.D.C., W.M.d.V. and A.E. are inventors on patent applications dealing with the use of *A. muciniphila* and its components in the treatment of metabolic disorders.
