# Supplementary material for: Food Reward Alterations during Obesity Are Associated with Inflammation in the Striatum in Mice: Beneficial Effects of Akkermansia muciniphila
Source: Cells. 2022 Aug 16;11(16):2534. doi: 10.3390/cells11162534 (PMC9406832; doi:10.3390/cells11162534)
Supplement: Supplementary file 1 [file cells-11-02534-s001.zip › cells-1839929-supplementary.pdf]

cells

MDPI

Supplementary materials

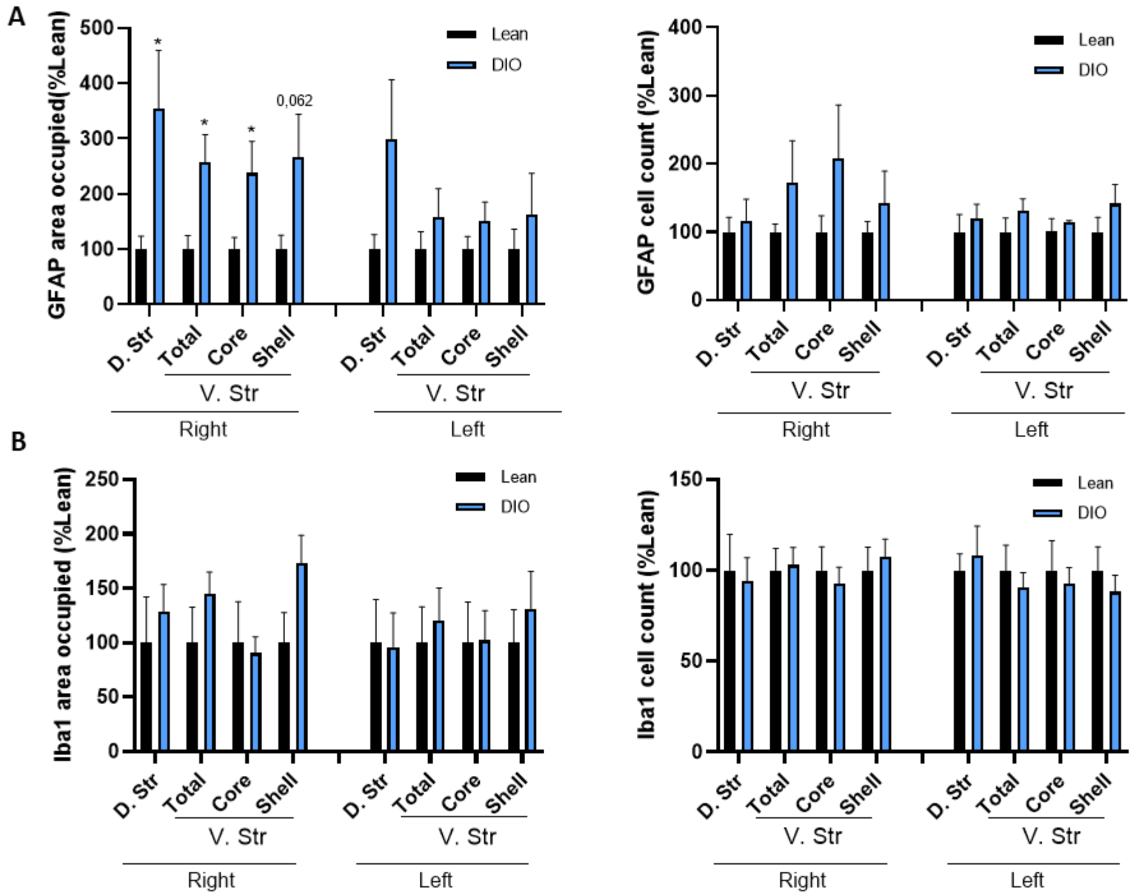

Figure S1. Details of immunofluorescence quantification. (A) Immunofluorescence quantification of the area occupied by astrocytes cells and the Gfap + cells in the right and left dorsal striatum (D. Str), total ventral striatum (V. Str total), ventral striatum core (V. Str core) and ventral striatum shell (V. Str shell) of lean and DIO mice (n=4-5/group). (B) Immunofluorescence quantification of the area occupied by microglial cells and the Iba1 + cells in the right and left dorsal striatum (D. Str), total ventral striatum (V. Str total), ventral striatum core (V. Str core) and ventral striatum shell (V. Str shell) of lean and DIO mice (n=5/group). Data are shown as mean ± SEM. P-values were obtained after unpaired Student's t-test or non-parametric Mann-Whitney test. *: p-value <0,05 between lean vs DIO.

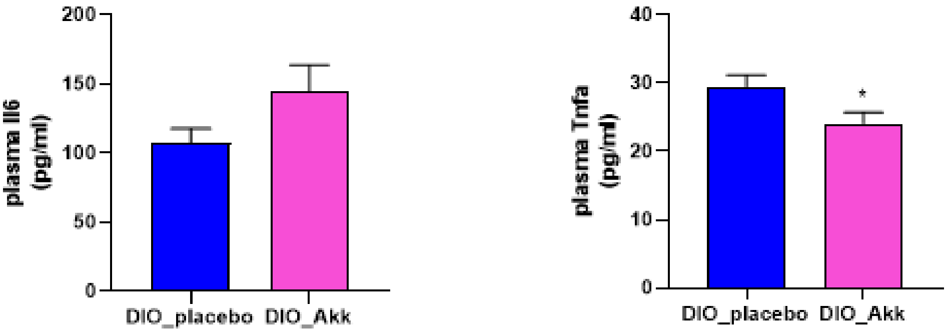

Figure S2. *Akkermansia muciniphila* administration reduces systemic inflammation. (A) Plasma concentration of the cytokines tumor necrosis factor alpha (Tnfa) and (B) interleukin-6 (Il6) measured by plasma multiplex analysis in mice treated with placebo (DIO_placebo) and *A. muciniphila* (DIO_Akk) (n=7-9/group). Data are shown as mean ± SEM. P-values were obtained after unpaired Student's t-test or non-parametric Mann-Whitney test. *: p-value < 0,05 between DIO_placebo vs DIO_Akk.

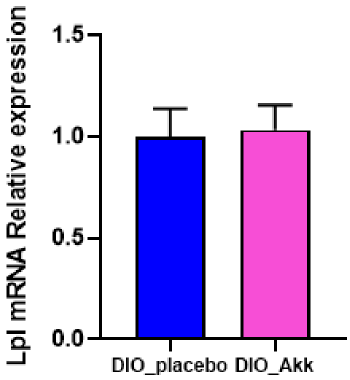

Figure S3. *Akkermansia muciniphila* administration do not influence *Lpl* expression in the jejunum. mRNA relative expression in the jejunum of lipoprotein lipase (*Lpl*) measured by qPCR in DIO mice treated with placebo (DIO_placebo) and *A. muciniphila* (DIO_Akk). (n=9/group). Data are shown as mean ± SEM. P-values were obtained after unpaired Student's t-test.

Table S1. Primer sequences.

Target gene	Forward primer sequence (5′–3′)	Reverse primer sequence (5′–3′)
Rpl19	GAAGGTCAAAGGGAATGTGTTCA	CCTGTCTGCCTTCAGCTTGT
Drd2	CCAAGAACGTGAGGGCTAAG	TGAGGATGCGAAAGGAGAAG
Drd1	GAGCCAACCTGAAGACACC	TGACAGCATCTCCATTTCCAG
Th	GCCAAGGACAAGCTCAGGAAC	ATCAATGGCCAGGGTGTACG
Dat	AAATGCTCCGTGGGACCAATG	GTCTCCCGCTCTTGAACCTC
Iba1	TCCTCGATGATCCCAAATACA	GTTTCTCCAGCATTCGCTTC
Tlr4	CCCTCAGCACTCTTGATTGC	TGCTTCTGTTCCTTGACCCA
Tlr2	CACCACTGCCCGTAGATGAA	GCCTCGGAATGCCAGCTT
Gfap	GCTCCAAGATGAAACCAACC	CCAGCGATTCAACCTTTCTC
Cd45 (Ptprc)	TGAGCACAACAGAGAATGCCC	AACACACCTGGATGATATGTGGT
Il1b	TCGCTCAGGGTCACAAGAAA	CATCAGAGGCAAGGAGGAAAAC
Tnfa	TCGAGTGACAAGCCTGTAGCC	TTGAGATCCATGCCGTTGG
Il6	ACAAGTCGGAGGCTTAATTACACAT	TTGCCATTGCACAACTCTTTTC
Cldn1	TTCGCAAAGCACCGGGCAGATACA	GCCACTAATGTCGCCAGACCTGAAA
Cldn5	TCATTGACCGGGAAGCTGAA	CCGGTGTCACAGAAGTACGA
Zo1	TTTTTGACAGGGGGAGTGG	TGCTGCAGAGGTCAAAGTTCAAG
Ocln	ATGTCCGGCCGATGCTCTC	TTTGGCTGCTCTTGGGTCTGTAT
Ccl2	GCAGTTAACGCCCCACTCA	TCCAGCCTACTCATTGGGATCA
Lpl	AGACTCGCTCTCAGATGCCCT	GCTTGCCATCCTCAGTCCC
Pnpla2	ACAGCTCCACCAACATCCAC	AGCCCTGTTTGCACATCTCT
